# Schottky Junctions with Bi Cocatalyst for Taming Aqueous Phase N_2_ Reduction toward Enhanced Solar Ammonia Production

**DOI:** 10.1002/advs.202003626

**Published:** 2021-01-31

**Authors:** Yewei Huang, Yisong Zhu, Shuijiao Chen, Xiuqiang Xie, Zhenjun Wu, Nan Zhang

**Affiliations:** ^1^ College of Materials Science and Engineering Hunan University Changsha 410082 P. R. China; ^2^ College of Chemistry and Chemical Engineering Hunan University Changsha 410082 P. R. China

**Keywords:** Bi, N_2_ adsorption, N_2_ activation, photocatalysis, Schottky junctions, solar ammonia production

## Abstract

Solar‐powered N_2_ reduction in aqueous solution is becoming a research hotspot for ammonia production. Schottky junctions at the metal/semiconductor interface have been effective to build up a one‐way channel for the delivery of photogenerated electrons toward photoredox reactions. However, their applications for enhancing the aqueous phase reduction of N_2_ to ammonia have been bottlenecked by the difficulty of N_2_ activation and the competing H_2_ evolution reaction (HER) at the metal surface. Herein, the application of Bi with low HER activity as a robust cocatalyst for constructing Schottky‐junction photocatalysts toward N_2_ reduction to ammonia is reported. The introduction of Bi not only boosts the interfacial electron transfer from excited photocatalysts due to the built‐in Schottky‐junction effect at the Bi/semiconductor interface but also synchronously facilitates the on‐site N_2_ adsorption and activation toward solar ammonia production. The unidirectional charge transfer to the active site of Bi significantly promotes the photocatalytic N_2_‐to‐ammonia conversion efficiency by 65 times for BiOBr. In addition, utilizing Bi to enhance the photocatalytic ammonia production can be extended to other semiconductor systems. This work is expected to unlock the promise of engineering Schottky junctions toward high‐efficiency solar N_2_‐to‐ammonia conversion in aqueous phase.

## Graphical abstract

Bi nanoparticles with low HER activity as the cocatalyst for constructing Schottky junctions toward enhancing photocatalytic N_2_‐to‐ammonia conversion have been reported, which can promote the photocatalytic N_2_‐to‐ammonia conversion efficiency by 65 times for BiOBr. The viability of Bi for boosting the photocatalytic N_2_‐to‐ammonia conversion in aqueous phase has also been confirmed in the TiO_2_ system.

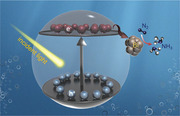

## 1 Introduction

Photocatalytic reduction of N_2_ offers a sustainable and distributed technology for ammonia production compared to the traditional Haber–Bosch process to complement the global nitrogen cycle, which has spurred enormous interest recently^[^ ^1^ ^]^. However, there are still fundamental factors restricting the solar‐to‐ammonia conversion efficiency over a single‐component photocatalyst^[^ ^2^ ^]^. On one hand, it generally suffers from high recombination rate of photogenerated charge carriers and insufficient interfacial transfer efficiency, which conspicuously limits the continuous and real‐time supply of electrons for the multi‐electron participated N_2_ reduction pathways. Another critical concern closely correlates to the intrinsic inertia of N_2_ molecules due to the high dissociation energy of N≡N bond (941 kJ mol^−1^), the absence of permanent dipole, and a high ionization potential (15.0 eV)^[^ ^3^ ^]^. It still demands breakthroughs in the fabrication of innovative photocatalysts featuring a dual functionality that not only steers electron flow, but also serves as N_2_ activation sites to achieve satisfying solar‐to‐ammonia conversion efficiency before it can be considered for industrial implementation. Toward this end, multifarious strategies have been exploited to ameliorate the activity of photocatalysts for N_2_ reduction reaction (NRR). This encompasses dopant/defect engineering^[^ ^4^ ^]^, selective crystal facets exposure^[^ ^5^ ^]^, and heterostructure construction by coupling two semiconductors^[^ ^6^ ^]^, etc.

A rectifying contact between semiconductor and metal with a low‐lying Fermi level (*E*_F_) leads to the formation of Schottky junction at the contacted interface, which could facilitate the photogenerated charge separation and trap the photogenerated electrons on metal. It is thus of great interest to investigate the possibility of applying Schottky junctions pumping electrons to enhance the photocatalytic activity for N_2_‐to‐ammonia conversion. Notably, apart from the application of Schottky junctions for enhancing gas phase N_2_ fixation with H_2_ as the reducing agent^[^ ^7^ ^]^, this Schottky effect has been less investigated for enhancing the efficiency of aqueous phase N_2_ reduction. Earlier case studies mainly focused on TiO_2_‐based photocatalysts, where the high density of states (*d*‐orbital) in the conduction band endows TiO_2_ with an excellent electron‐accepting ability for N_2_ chemisorption by electron back‐donation from TiO_2_ to antibonding π* orbitals of N_2_ and the loaded metals (denoted as M) catalyze the hydrogen dissociation into H_ad_ to form M‐H_ad_^[^ ^8^ ^]^. However, the Schottky effect directed charge transfer leads to the electron depletion at TiO_2_, which is disadvantageous for N_2_ chemisorption dominated by electron transfer. On the other hand, the M‐H_ad_ strength is generally weak for most metals^[^ ^8^, ^9^ ^]^, which preferably results in proton reduction and concomitant H_2_ evolution as an undesirable side reaction in aqueous solution, thereby limiting the NRR selectivity. Consequently, research on utilizing M‐semiconductor Schottky junction to promote the aqueous phase photocatalytic N_2_ reduction is still scarce and limited in the scope.

According to the theoretical investigations by Norskov and coworkers, the hydrogen binding energy (Δ*G*_H_^*^) over Bi is calculated to be ≈0.75 eV, which is among the highest for a library of elements, including those widely used for constructing Schottky‐junction photocatalysts, such as Pt, Pd, and Ag^[^ ^8^, ^9^ ^]^. The high ΔG_H_^*^ value indicates the low HER‐activity of Bi and has been proved experimentally^[^ ^9^ ^]^. On the other hand, bismuth‐based compounds have received considerable interest for photocatalytic N_2_‐to‐ammonia conversion^[^ ^4^, ^10^ ^]^. For example, Zhang and co‐workers demonstrated that BiOBr nanosheets with oxygen vacancies (OVs) on the surface can significantly improve the adsorption and activation of N_2_, resulting in the successful photocatalytic N_2_‐to‐ammonia conversion^[^ ^4a^ ^]^. It is found that the chemisorbed N_2_ molecules preferably coordinate with Bi atoms with an end‐on bound structure, and the back electron transfer to N_2_ activates the N≡N bond, resulting in an increase in the bond length from 1.078 Å in the original N_2_ to 1.133 Å. In addition, theoretical investigations showed that Bi‐terminated facets possessed more active centers than O‐terminated facets^[^ ^11^ ^]^, further demonstrating the favorable N_2_ adsorption and activation over Bi sites. These fundamentals of Bi well meet the criteria of ideal metal cocatalyst desired for constructing Schottky junctions for photocatalytic N_2_‐to‐ammonia conversion as aforementioned, which motivate us to focus on elemental Bi to validate its viability for improving the photocatalytic NRR performance in aqueous phase via Schottky effect. Herein, by using BiOBr as a semiconducting scaffold, Bi nanoparticles (NPs) have been deposited to construct typical Schottky‐junction photocatalysts. Encouragingly, it is found that the ammonia production rate is enhanced 65 times over Bi/BiOBr compared to bare BiOBr without detectable hydrogen evolution in water. A series of control experiments have been conducted, which demonstrate the critical role of Bi in steering the unidirectional photogenerated electron flow and simultaneously activating N_2_ for enhancing solar ammonia production. In addition, the capability of Bi in enhancing the photocatalytic ammonia production can be also confirmed in other semiconductor systems. It is expected that our exploration could provide inspirational insights to boost solar‐to‐ammonia conversion efficiency by elaborated materials engineering.

## 2 Results and Discussion

**Figure 1:**
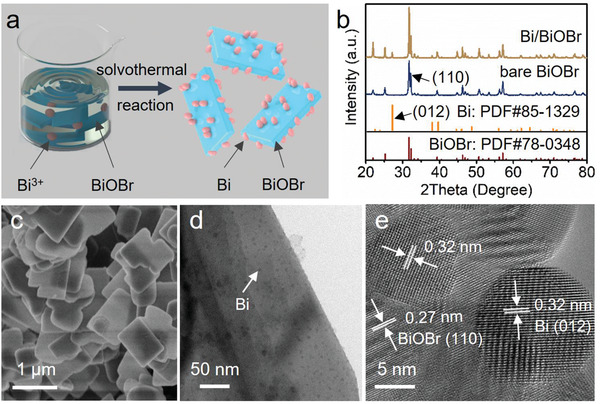
a) Schematics of the preparation of Bi/BiOBr composites. b) XRD patterns of bare BiOBr and Bi/BiOBr. c) SEM image Bi/BiOBr. d) TEM and e) HRTEM images of Bi/BiOBr.

We have deliberately prepared the negatively charged BiOBr nanoplates as the photoactive semiconductor for the construction of the target Bi‐based Schottky‐junction composite photocatalysts. The zeta potential of the as‐prepared BiOBr has been measured to be ‐13.5 mV (Figure S1, Supporting Information). As can be seen from Figure S2 (Supporting Information), the obtained BiOBr features an irregular sheet‐like structure. Then, Bi(NO_3_)_3_ was added into the BiOBr suspension to serve as the precursor of metallic Bi. As schematically shown in **Figure** **1**a, loading of Bi NPs onto BiOBr nanoplates has been realized by a solvothermal method using glycol as a reducing agent to reduce the Bi^3+^. In such reaction system, the electrostatic interactions between cationic Bi^3+^ and negatively charged BiOBr facilitate the nucleation and growth of Bi onto BiOBr. The concentration of the residual Bi^3+^ in the solvent after this solvothermal treatment for the preparation of Bi/BiOBr with the highest Bi ratio of 5 wt% in the present study has been quantified by an inductively coupled plasma emission spectroscopy instrument. The value is determined to be 2 µg mL^−1^, which is almost negligible compared to that before the solvothermal treatment (300 µg mL^−1^), suggesting that Bi^3+^ has been successfully converted to Bi nanoparticles loaded on BiOBr by the solvothermal reaction and the ratio of Bi in the resulting Bi/BiOBr composites is nearly identical to the nominal value.

The crystalline phase and composition of the samples were investigated by X‐ray diffraction (XRD) patterns (Figure 1b). All diffraction peaks of the bare BiOBr can be indexed to pure tetragonal BiOBr phase (JCPDS no. 78–0348). For the resulting Bi/BiOBr, additional diffraction peaks originating from Bi are discernable, indicating the successful preparation of Bi/BiOBr composites. X‐ray photoelectron spectroscopy (XPS) has been conducted to further investigate the composition of the samples. As shown in Figure S3 (Supporting Information), the Bi 4f spectrum of bare BiOBr presents a typical spin‐orbit doublet splitting centered at 159.7 and 165.0 eV, corresponding to Bi^3+^ in BiOBr without oxygen vacancies (OVs)^[^ ^12^ ^]^. For the Bi/BiOBr composite, two additional peaks located at 157.6 and 163.1 eV are observed, corresponding to a 1.9 eV red shift relative to the peaks of Bi^3+^ in BiOBr. These peaks can be attributed to the binding energies of Bi 4f_7/2_ and Bi 4f_5/2_ in metallic Bi, which further confirm the formation of Bi NPs. To further probe surface OVs of the samples, electron spin resonance (ESR) spectra have been collected. As shown in Figure S4 (Supporting Information), no signal of OVs is observed in the ESR spectra of the bare BiOBr and Bi/BiOBr samples, indicating the absence of OVs. The morphology of Bi/BiOBr composites has been investigated by scanning electron microscopy (SEM). As shown in Figure 1c, Bi/BiOBr presents a typical plate‐like structure with a length of 1–2 µm. Figure 1d shows the transmission electron microscopy (TEM) image of Bi/BiOBr. It can be seen that Bi nanoparticles are uniformly loaded on the BiOBr substrate with intimate interface interactions, which is beneficial for interfacial charge transfer. We analyzed the size distribution of Bi nanoparticles (Figure S5, Supporting Information). Accordingly, the average diameter of Bi nanoparticles is 4.0 nm. Figure 1e shows the high‐resolution transmission electron microscopy (HRTEM) image of Bi/BiOBr, in which the interlayer spacing of 0.32 nm is observed, corresponding to the lattice constant of Bi (012) planes^[^ ^13^ ^]^. On the other hand, the measured lattice spacing of 0.27 nm corresponds to the (110) crystal plane of BiOBr.

**Figure 2:**
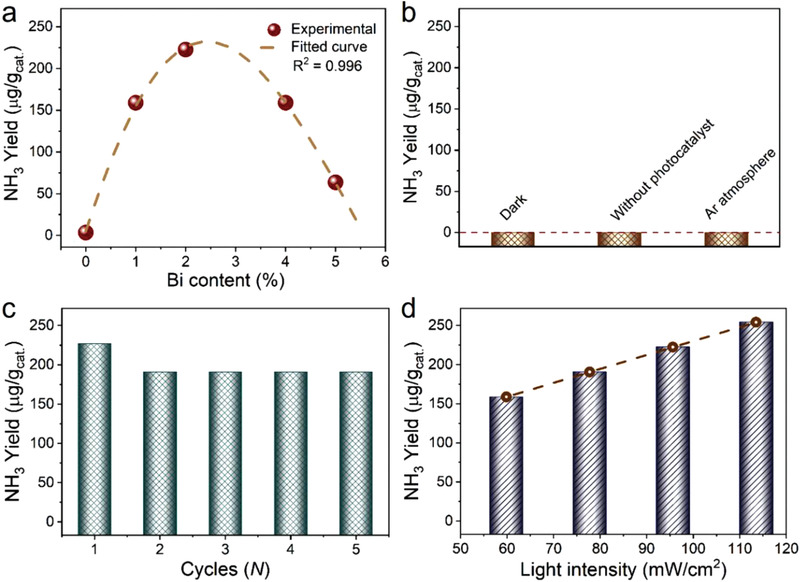
a) Dependence of photocatalytic ammonia production on the Bi content in Bi/BiOBr composites under visible light irradiation (*λ* ≥ 420 nm) in N_2_ atmosphere. b) Controlled experiments for photocatalytic ammonia production over Bi/BiOBr under different conditions. c) Photostability of Bi/BiOBr composites for photocatalytic ammonia production. d) Photocatalytic ammonia production over Bi/BiOBr under visible light (*λ* ≥ 420 nm) with varied irradiation intensity.

Next, we have investigated the photoactivities of the samples for N_2_‐to‐ammonia conversion under visible light irradiation (*λ* ≥ 420 nm) using Na_2_SO_3_ as the hole scavenger. The generated NH_3_ was spectrophotometrically measured based on indophenol blue method (Figure S6, Supporting Information). In our experiments, a trace amount of ammonia has been detected before the photocatalytic reaction (0.05 µg mL^−1^, Figure S7, Supporting Information). To avoid experimental artifacts interfering the results and have a reliable analysis of photocatalytic N_2_‐to‐ammonia conversion, the background ammonia has been subtracted when quantifying the photocatalytically produced ammonia in the present investigations. **Figure** **2**a shows the produced ammonia amount over Bi/BiOBr catalysts with different contents of Bi loadings, together with that over bare BiOBr for comparison. Only trace amount of ammonia (3.4 µg g_cat._^−1^) has been detected over bare BiOBr, suggesting that N_2_ activation sites are indispensable for BiOBr. After loading 1.0 wt% of Bi on BiOBr, the activity in N_2_‐to‐ammonia conversion is significantly increased and generated ammonia reaches up to 159.0 µg g_cat._^−1^. With the increase of the amount of Bi loaded on BiOBr, the ammonia evolution efficiency on Bi/BiOBr is increased further and achieves a maximum when the loading amount of Bi on BiOBr is about 2.0 wt%. The produced ammonia is increased to 222.6 µg g_cat._^−1^ after loading 2.0 wt% of Bi on BiOBr, which is about 65 times as high as that of bare BiOBr. The dependence of photoactivity on the Bi content in Bi/BiOBr composites exhibits a volcano‐type curve and a further increase of Bi content leads to the decrease of photocatalytic ammonia production. Notably, these Bi/BiOBr samples have similar surface areas (Table S1, Supporting Information), indicating that the surface area difference is unlikely the major factors leading to the large discrepancy in their photoactivities. Figure S8 (Supporting Information) shows the XRD patterns of the Bi/BiOBr composites with different Bi ratios and the FWHM (full width at half‐maximum) values of Bi (012) diffraction peak for Bi/BiOBr composites are summarized in Table S2 (Supporting Information). It can be seen that the FWHM values are similar, suggesting the particle size of Bi in the Bi/BiOBr composites are statistically identical according to the Scherrer's equation. To investigate the dependence of photoactivity on the loading ratio of Bi in Bi/BiOBr composites, we tested transient photocurrent responses of the Bi/BiOBr composites under visible light irradiation (*λ* ≥ 420 nm). As can be seen in Figure S9 (Supporting Information), the photocurrent of 2% Bi‐BiOBr is higher than that of 1% Bi‐BiOBr and bare BiOBr because the introduction of Bi facilitates the separation of photogenerated electrons and holes. However, the photocurrent of 4% Bi‐BiOBr is lower than that of 2% Bi‐BiOBr. When the loading ratio of Bi increases to 5%, the photocurrent further decreases. The trend of photocurrent responses is in consistence with the photoactivities. Consequently, the appearance of a maximum in activity with an optimum loading of cocatalyst could be ascribed to that an excess amount of metal cocatalyst acts as the combination center of photogenerated charge carriers^[^ ^14^ ^]^, thereby limiting electrons available for NRR. The photocatalytic ammonia synthesis activity of the 2% Bi‐BiOBr photocatalyst in our present work is comparable to some state‐of‐the‐art systems (Table S3, Supporting Information). In the following studies, the optimal Bi/BiOBr with Bi loading ratio of 2.0 wt% is selected as the typical interest.

As shown in Figure 2b, for the experiments without photocatalysts or in the dark, no ammonia production has been detected, suggesting the photocatalysis nature of the N_2_‐to‐ammonia conversion over the Bi/BiOBr composites. Furthermore, Bi/BiOBr exhibited no activity in Ar atmosphere under light irradiation. These joint controlled experiments unambiguously indicate that the ammonia production shown in Figure 2a originates from the reduction of the supplied N_2_ gas rather than contaminations from either N_2_ flow or the possible reduction of residual NO_3_^−^ in the solution, which is always a concern of researchers in this area. Further discussion on the nitrogen source of the detected ammonia can be found in the Note S1 (Supporting Information). The apparent quantum efficiencies (AQEs) of the 2% Bi‐BiOBr composite photocatalysts have been measured under diverse monochromatic light irradiation. The AQE at *λ*  = 420 nm is calculated to be 0.23%, while the AQE is almost negligible at the wavelength of 605 nm. The result in Figure 2c demonstrates that Bi/BiOBr composites exhibit good cycling stability over successive five photocatalytic cycles. As shown in Figure S10 (Supporting Information), the diffraction peaks of Bi nanoparticles are obviously discernable and no additional phase can be observed after cycling. We further supplemented the TEM images of the cycled Bi/BiOBr composite. From Figure S11 (Supporting Information) it can be seen that Bi nanoparticles can be observed, which is in agreement with the XRD result. The deactivation of Bi/BiOBr could be ascribed to the photocorrosion of BiOBr via oxidative consumption of lattice Br according to the XPS results (Figure S12, Supporting Information). Notably, a prolonged irradiation time would lead to the decrease of ammonia yield (Figure S13, Supporting Information), which has been also observed over BiOCl photocatalysts due to the oxidative decomposition of the accumulated NH_3_ reported by Shiraishi and coworkers^[^ ^15^ ^]^. This issue can be potentially alleviated by optimizing the reaction medium using acidic bromide solutions^[^ ^15^ ^]^ or applying a flow reaction system.

To reveal the role of Bi in the enhancement of the photocatalytic NRR performance of Bi/BiOBr composites, a series of characterizations have been performed comparatively. First, the band structure of BiOBr has been analyzed to facilitate the understanding of the separation, transfer, and recombination process of the photogenerated charge carriers over Bi/BiOBr composites. Figure S14a (Supporting Information) shows the optical absorption property of BiOBr, which exhibits distinct absorption with an edge at around 435 nm, corresponding to the band gap absorption of BiOBr component. The band gap of BiOBr is measured by Kubelka‐Munk plots converted from the absorption spectrum, which is 2.7 eV (Figure S14b, Supporting Information). Figure S15 (Supporting Information) shows the Mott–Schottky (MS) plot of the BiOBr electrode. The positive slope of the C^−2^−E plot suggests the n‐type semiconductor of BiOBr^[^ ^16^ ^]^. Accordingly, the flatband potential of BiOBr is determined to be −0.46 V (vs normal hydrogen electrode, NHE). On the basis of the band‐gap values obtained above and the relationships among energy levels of n‐type semiconductors (the detailed discussion can be found in the Supporting Information), the potentials of conduction band (*E*_CB_) and valence band (*E*_VB_) are −0.46 and 2.24 V (vs NHE), respectively (Figure S16, Supporting Information). The work function (*φ*) of Bi is 4.36 eV (vs absolute vacuum scale, AVS)^[^ ^17^ ^]^, corresponding to a Fermi level (*E*_F_) of −0.14 V (vs NHE). Consequently, the photogenerated electrons thermodynamically transfer to Bi driven by the Schottky‐junction effect (Figure S16, Supporting Information), which is favorable for the separation of photogenerated charge carriers, thereby facilitating NRR. The room‐temperature photoluminescence (PL) spectra have been applied to investigate the recombination of photogenerated electron−hole pairs in the as‐prepared materials. As shown in Figure S17 (Supporting Information), Bi/BiOBr exhibits lower PL intensity than that of bare BiOBr, further confirming that the introduction of Bi improves the charge separation efficiency of BiOBr. Notably, although photocatalytic HER is thermodynamically feasible in the present system (Figure S16, Supporting Information), no H_2_ has been detected over either bare BiOBr or Bi/BiOBr composites (Figure S18, Supporting Information), which should be ascribed to the large HER overpotentials. Our results are in agreement with the reports showing that Bi has low HER‐activity^[^ ^8^, ^9^ ^]^.

We have then investigated the possible contribution of Bi surface plasmon resonance (SPR) effect to the photoactivity enhancement of Bi/BiOBr composites. Although the absorption of Bi/BiOBr composites in the visible‐light region intensified as compared to bare BiOBr, no obvious SPR peak in the region of 400–800 nm can be observed as the content of Bi increased (Figure S19, Supporting Information), excluding the plasmonic excitation of Bi in the visible light region. It has been exemplified that the SPR effect of Bi closely depends on the size and shape of Bi NPs as well as the dielectric constant of the environment^[^ ^18^ ^]^. Our observation is in agreement with the previous report by Foos et al., which indicates that the SPR of Bi NPs with diameters ranging from 3.2 to 8.0 nm appeared at wavelength less than 196 nm^[^ ^19^ ^]^. Furthermore, it has been reported that in plasmonic metal‐semiconductor composites, when the intensity of light irradiation to excite both components was increased to some extent, the photocatalytic efficiency was lowered due to the formation of two electron flows in reverse directions^[^ ^20^ ^]^. In this regard, to further study whether the SPR effect of Bi NPs enhances the photoactivity of Bi/BiOBr toward NRR, we have evaluated the photocatalytic performance of the composites under different irradiation intensities. As shown in Figure 2d, the produced ammonia amounts over Bi/BiOBr increase almost linearly as the light intensity is enhanced. Consequently, it is reasonable to exclude the possibility of the Bi SPR effect contributing to the photoactivity enhancement of the Bi/BiOBr Schottky‐junction photocatalysts and the unidirectional electron flow from the photoexcited BiOBr to Bi can be established.

**Figure 3:**
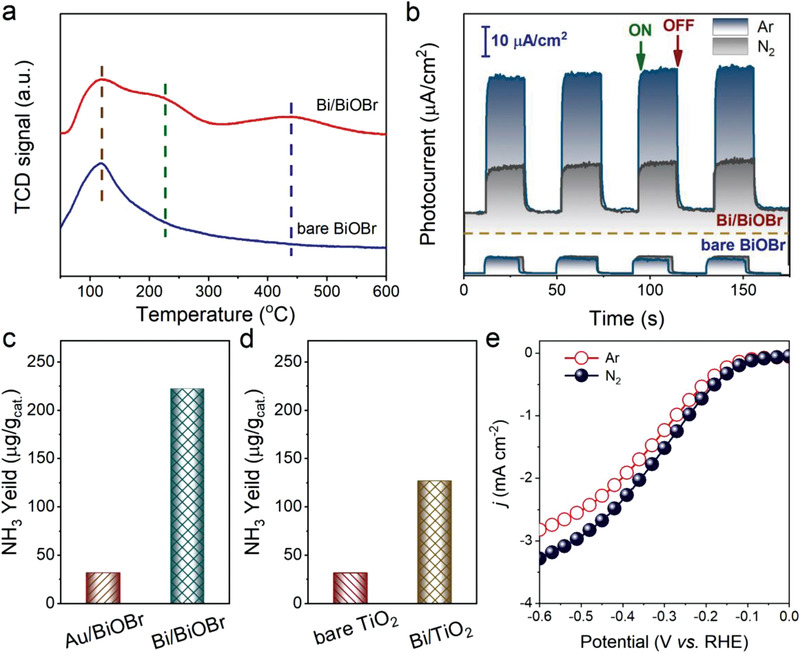
a) N_2_‐TPD profiles of bare BiOBr and Bi/BiOBr composites. b) Transient photocurrent responses of bare BiOBr and Bi/BiOBr under visible light at Ar and N_2_ atmospheres without bias. c) Comparison of NH_3_ production over Au/BiOBr and Bi/BiOBr. d) Photoactivities of bare TiO_2_ and Bi/TiO_2_ under UV–vis irradiation (*λ* ≥ 365 nm). e) Linear sweep voltammetric curves in N_2_ saturated (blue line) and Ar saturated (red line) electrolytes.

To gain a fundamental insight into the role of Bi in the N_2_ adsorption and activation for the photocatalytic NRR performance enhancement of Bi/BiOBr composites, temperature‐programmed desorption of N_2_ (N_2_‐TPD) was used to investigate N_2_ adsorption over the photocatalyst surface. As shown in **Figure** **3**a, a desorption peak of N_2_ centered at around 120 °C can be observed for both bare BiOBr and Bi/BiOBr composite, corresponding to the physical adsorption of N_2_ over BiOBr^[^ ^2d^ ^]^. Compared to bare BiOBr, additional desorption peaks at around 240 and 450 °C have been clearly observed for Bi/BiOBr. This result is similar to the adsorption of N_2_ over BiO^[^ ^21^ ^]^, which indicates the excellent N_2_ chemisorption. The N_2_ TPD results suggest the indispensable role of Bi in the surface chemisorption of N_2_. Transient photocurrent responses of bare BiOBr and Bi/BiOBr at Ar and N_2_ atmospheres further verify the predominating role of Bi NPs in activating N_2_. As can be seen from Figure 3b, the transient photocurrent response of bare BiOBr at N_2_ atmosphere is almost identical to that at Ar inert atmosphere, indicating the interfacial electron transfer of bare BiOBr was not affected in the presence of N_2_. In contrast, the photocurrent density of Bi/BiOBr composites under N_2_ atmosphere is lower than that at Ar atmosphere. This could be ascribed to the reduction reaction of N_2_ over Bi, which consumes photogenerated electrons, thereby reducing the proportion of electrons transferring to the counter electrode through the external circuit^[^ ^4a^ ^]^.

To gain the kinetics information of the photocatalytic N_2_‐to‐ammonia conversion over Bi/BiOBr composites, Au/BiOBr composite with an identical metal loading ratio to that of Bi/BiOBr (2 wt%) has been prepared (Figure S20, Supporting Information) and tested for photocatalytic NRR. It is known that Au has a higher work function of 4.78 eV than that of Bi^[^ ^17^ ^]^. As a result, Au should be more effective than Bi in terms of promoting the separation of photogenerated charge carriers. This has been verified by the lower PL intensity of Au/BiOBr than that of Bi/BiOBr counterparts (Figure S17, Supporting Information). However, Au/BiOBr shows decreased photoactivities for ammonia production compared to Bi/BiOBr (Figure 3c), suggesting that N_2_ activation rather than photogenerated charge carriers separation is the rate‐limiting step for photocatalytic NRR. Furthermore, it manifests the promise of Bi to build Schottky‐junction photocatalysts and, more importantly, taming the activation of N_2_ toward solar ammonia production. The feasibility of Bi‐enhanced photocatalytic ammonia production has been further confirmed in the TiO_2_ system. Bi loaded TiO_2_ (Bi/TiO_2_) has been successfully prepared and confirmed by XRD (Figure S21, Supporting Information). As shown in Figure 3d, the photocatalytic ammonia production over Bi/TiO_2_ is 4 times higher than that over bare TiO_2_. Linear sweep voltammetric (LSV) curves for Bi/TiO_2_ composites have been conducted and the results are presented in Figure 3e. Enhancement of the current density has been achieved in N_2_‐saturated solutions compared to the Ar‐saturated, which could be taken as a further evidence that Bi NPs are efficient for the activation of N_2_. Notably, similar cathodic scan for BiOBr‐based systems is bottlenecked by the electrochemical reduction of BiOBr to Bi (Figure S22, Supporting Information), as has also been confirmed by the previous study^[^ ^22^ ^]^.

In combination with the aforementioned discussions, the pivotal role of Bi in enhancing the photoactivity of Bi/BiOBr toward N_2_‐to‐ammonia conversion is two‐fold. On one hand, due to the built‐in Schottky‐junction effect at the Bi/BiOBr interface, the separation efficiency of photogenerated charge carriers of BiOBr is enhanced by the introduction of Bi NPs. On the other hand, Bi NPs with low HER‐activity facilitate the surface adsorption and reduction of N_2_ toward solar ammonia production while minimizing the competing HER. The synergy of these two factors results in the unidirectional charge transfer to the active site of Bi NPs, which significantly promotes the photocatalytic N_2_‐to‐ammonia conversion.

**Figure 4:**
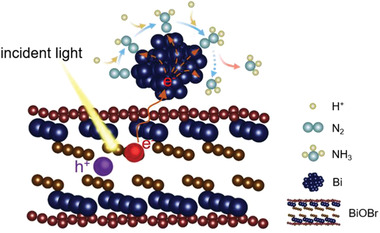
Schematics of photocatalytic N_2_‐to‐ammonia conversion over Bi/BiOBr composites.

We have further tried to understand the reaction pathway of the photocatalytic N_2_‐to‐ammonia conversion over Bi/BiOBr composites on the molecular level. The dissociative mechanism of N_2_ reduction to NH_3_ requires harsh conditions such as high‐pressure and high‐temperature for the N≡N triple bond cleavage, which normally occurs in the industrial Haber−Bosch reaction^[^ ^23^ ^]^. Consequently, two different associative N_2_ hydrogenation pathways over Bi/BiOBr composite photocatalysts have been considered, i.e., alternating pathway and distal pathway. The theoretical investigation by Zhang and coworkers showed that N_2_ preferably adsorb on BiOBr with oxygen‐vacancies through coordinating with the partially reduced Bi cations with an end‐on bound structure^[^ ^4a^ ^]^. We thus envisage the similar end‐on surface adsorption configuration of N_2_ on the surface of Bi NPs, as depicted in **Figure** **4**. In the alternating pathway, the two nitrogen atoms experience alternate hydrogenation, which however might lead to the production of a variable amount of hydrazine due to the possible shunts from diazene and/or hydrazine^[^ ^24^ ^]^. In contrast, in the distal pathway, the remote N atom of N_2_ on the surface of is hydrogenated successively until NH_3_ is released and the addition of protons at the surface N atoms occurs. Following this pathway, only NH_3_ is produced, and no other byproducts will be observed^[^ ^25^ ^]^. We have tested hydrazine by the colorimetric method (Figure S23, Supporting Information). However, no hydrazine has been detected during the photocatalytic NRR over Bi/BiOBr composites (Figure S24, Supporting Information). This preliminary result suggests that the hydrogenation of N_2_ over Bi/BiOBr could follow the distal pathway, and NH_3_ is produced with the photogenerated electrons accumulated on Bi NPs (Figure 4). In addition, ion chromatography analysis has not detected photocatalytically produced NO_3_^−^ or NO_2_^−^ (Figure S25, Supporting Information).

## 3 Conclusions

In summary, we proposed Bi NPs with low HER‐activity as the cocatalyst for constructing Schottky junctions toward enhancing photocatalytic N_2_‐to‐ammonia conversion. The crucial role of Bi NPs in controlling electron transfer and activating N_2_ has been revealed. It is found that Bi NPs effectively accumulate photogenerated electrons from the semiconductor photocatalysts due to the Schottky‐junction effect. In addition, Bi NPs contribute to accelerate the critical rate‐limiting step of N_2_ surface adsorption and activation. The bifunctionality of Bi NPs steers the unidirectional electron flow to the exposed active sites for NRR, thereby synergistically promoting solar ammonia production efficiency in aqueous phase. Our investigation is expected to be an inspiration source in tapping the potential of metal‐semiconductor hybrids to realize their efficient exploitation for photocatalytic N_2_‐to‐ammonia conversion in aqueous phase. In addition, Bi‐based Schottky‐junction effect to enhance photocatalytic NRR is expected to be extended to other photocatalyst systems with high photon conversion efficiency and broadband light harvesting ability, such as those integrated with cocatalysts shuttling photogenerated holes and 2D ultrathin photocatalysts, thereby further promoting solar‐to‐ammonia conversion efficiency.

## 4 Experimental Section

Bismuth(III) nitrate pentahydrate (Bi(NO_3_)_3_·5H_2_O), potassium bromide (KBr), titanium(IV) oxide (TiO_2_), tetrachloroauric(III) acid tetrahydrate (HAuCl_4_·4H_2_O), sulfuric acid (H_2_SO_4_), and sodium sulfite (Na_2_SO_3_) were of analytical grade and supplied by Sinopharm Chemical Reagent Co. Ltd (Shanghai, China). An ultrapure water purification system (Eco‐S15Q, Hitech Instruments Co. Ltd, Shanghai, China) was used to produce 18.2 MΩ cm^−1^ deionized water (DI water) in all the experiments.

##### Synthesis of BiOBr

BiOBr flakes were obtained by a hydrolysis process^[^ ^26^ ^]^. Typically, 0.1190 g (1 mmol) of KBr was dissolved in 15 mL of DI water and sink in a 90 °C oil bath. 0.4858 g (1 mmol) of Bi(NO_3_)_3_·5H_2_O was dispersed in 20 mL of ethanol by ultrasonication for 10 min and then dropwise added in the KBr solution. The mixture was stirred for 3 h at 90 °C and then naturally cooled to room temperature. The precipitation was washed with DI water and ethanol several times and collected by centrifugation. The product was obtained after drying at 60 °C overnight.

##### Synthesis of Bi/BiOBr

120 mg of BiOBr was dispersed in 16 mL of anhydrous ethylene glycol and then 2 mL of ethylene glycol solution containing Bi(NO_3_)_3_·5H_2_O was added dropwise to the BiOBr dispersion under stirring. The mixture was stirred for another 1 h at room temperature and then transferred into a 20 mL Teflon‐lined stainless autoclave. The autoclave was allowed to be heated at 180 °C for 12 h under autogenous pressure, and then air cooled to room temperature. The resulting precipitates were collected and washed with deionized water and ethanol. The final product was dried at 60 °C for further use. Au/BiOBr composites were prepared in a similar way by using HAuCl_4_·4H_2_O as the Au precursor. In addition, Bi/TiO_2_ composites were prepared by a similar solvothermal method except that commercial TiO_2_ (Degussa P25) was used.

##### Characterization

The crystal phase of the sample was analyzed by XRD with an X‐ray diffractometer (Bruker D8 Advance diffractometer, Cu K*α*1). The concentration of Bi^3+^ was quantified by an inductively coupled plasma emission spectroscopy instrument (ICP, PerkinElmer Optima 2000DV). The morphology and structure of the products were characterized by SEM (Hitachi, S‐4800), TEM, and HRTEM (Titan G2 60–300 with image corrector). XPS was conducted on a ESCALAB Xi+. The ESR was conducted on a Bruker ESR A300 spectrometer. UV−vis diffuse reflectance spectra (DRS) were recorded on a UV−vis spectrometer (Shimadzu UV‐2550), using 100% BaSO_4_ as the reflectance sample. PL spectra were detected with an F‐7000 spectrophotometer (Hitachi, Japan), excited by an incident light of 280 nm. N_2_ adsorption–desorption isotherms were measured on a Quantachrome NOVA 4000e at 77 K. The specific surface areas (SSAs) were calculated by the Brunauer–Emmet–Teller (BET) equation. The photoelectrochemical analysis was carried out on an electrochemical station (CHI660D) in a conventional three‐electrode cell using a Pt plate and an Ag/AgCl electrode as the counter electrode and reference electrode, respectively. The working electrode was prepared by dip‐coating for transient photocurrent response and Mott‐Schottky experiments. Specifically, 5 mg of photocatalyst was mixed with 0.5 mL of N‐methyl pyrrolidone (NMP) to form a uniform photocatalyst‐NMP suspension in an ultrasonic bath. The suspension was then dropped onto an indium tin oxide (ITO) glass (10 mm × 50 mm) in a circular area with a diameter of 6 mm. The boundary of ITO glass was protected using Scotch tape. The Scotch tape was unstuck after drying overnight at 60 °C and the uncoated part of the electrode was isolated with epoxy resin. The electrolyte was the same as the photocatalysis experiment for photocurrent measurement.

##### Photocatalytic Activity Tests

25 mg of the photocatalyst was dispersed in 50 mL of a mixed solution of 0.025 m H_2_SO_4_ and 0.025 m Na_2_SO_3._ Nitrogen flow was continuously bubbled through the solution at a rate of 60 mL min^−1^. Before light irradiation, the suspension was bubbled under dark with magnetic stirring for 1 h. 1 mL of the obtained suspension was taken out after irradiation for 10 min, and the photocatalyst in the solution was filtered off with a needle filter. The concentration of ammonia in the solution was analyzed by indophenol blue method. The reaction conditions of the recycle test were the same as the above mentioned. After each cycle, the photocatalyst was thoroughly washed and collected by centrifugation. The photocatalytic hydrogen evolution was performed in a quartz reactor. In a typical photocatalytic experiment, 2.5 mg of the photocatalysts was dispersed in a 5 mL of a mixed solution of 0.025 m H_2_SO_4_ and 0.025 m Na_2_SO_3_. Prior to the light irradiation, the reaction solution was evacuated by a mechanical pump several times to remove air completely. The photocatalytic system was irradiated with visible light (*λ* ≥ 420 nm) by a 300 W Xe arc lamp (PLS‐SXE 300, Beijing Perfectlight). The photocatalytic reaction was typically performed for 4 h, and 1 mL of reactive gas was taken from the reactor with a syringe for analysis using a gas chromatograph (GC 2014C, 5 A molecular sieve column, TCD, Ar carrier). Nitrate and nitrite ions were analyzed by ion chromatograph (Thermo Scientific Dionex Aquion).

## Conflict of Interest

The authors declare no conflict of interest.

## Supporting information

Supporting Information
