# Supplementary material for: Schottky Junctions with Bi Cocatalyst for Taming Aqueous Phase N2 Reduction toward Enhanced Solar Ammonia Production
Source: Adv Sci (Weinh). 2021 Jan 31;8(6):2003626. doi: 10.1002/advs.202003626 (PMC7967041; doi:10.1002/advs.202003626)
Supplement: Supplementary file 1 — Supporting Information [file ADVS-8-2003626-s001.pdf]

© 2020 Wiley-VCH GmbH

ADVANCED SCIENCE
Open Access

Supporting Information

for *Adv. Sci.,* DOI: 10.1002/advs.202003626

Schottky Junctions with Bi Cocatalyst for Taming Aqueous Phase N_2 Reduction toward Enhanced Solar Ammonia Production

Yewei Huang, Yisong Zhu, Shuijiao Chen, Xiuqiang Xie, Zhenjun Wu*, Nan Zhang**

Supporting Information

Schottky Junctions with Bi Cocatalyst for Taming Aqueous Phase N_2 Reduction toward Enhanced Solar Ammonia Production

Yewei Huang, Yisong Zhu, Shuijiao Chen, Xiuqiang Xie, Zhenjun Wu*, Nan Zhang**

Contents list

Figure S1. Zeta potential of bare BiOBr.

Figure S2. SEM image of bare BiOBr.

Figure S3. Fitted XPS Bi 4f spectra of the as-prepared bare BiOBr and Bi/BiOBr composites.

Figure S4. ESR spectra of bare BiOBr and 2% Bi-BiOBr.

Figure S5. Size distribution histogram of Bi nanoparticles.

Figure S6. Calibration curve used for the qualification of NH_4^+ by indophenol blue method.

Figure S7. Spectrophotometric assay of the background ammonia by the indophenol blue method.

Figure S8. XRD patterns of bare BiOBr and Bi/BiOBr composites with different Bi ratios.

Figure S9. Transient photocurrent responses of the Bi/BiOBr composites under visible light irradiation ($\lambda \geq 420$ nm) without bias.

Figure S10. XRD patterns of 2% Bi-BiOBr composite before and after photocatalytic activity test.

Figure S11. (a) TEM and (b) HRTEM images of 2% Bi-BiOBr after 5 cycles of photocatalytic reaction.

Figure S12. Fitted XPS Bi 4f spectra of 2% Bi-BiOBr composites after photoactivity test.

Figure S13. Time dependent NH_3 yield profile of 2% Bi-BiOBr under visible light irradiation ($\lambda \geq 420$ nm) in N_2 atmosphere.

Figure S14. (a) DRS spectrum and (b) Tauc plot of $(\alpha h\nu)^{1/2}$ versus photon energy of bare BiOBr.

Figure S15. Mott–Schottky (MS) plots of BiOBr.

Figure S16. Schematic illustration of electron transfer for Bi/BiOBr composites; CB and VB are short for conduction band and valence band, respectively.

Figure S17. PL spectra of bare BiOBr, 2% Bi-BiOBr, and 2% Au-BiOBr.

Figure S18. Photocatalytic hydrogen evolution over bare BiOBr and 2% Bi-BiOBr under visible light irradiation (≥ 420 nm).

Figure S19. DRS spectra of bare BiOBr and Bi/BiOBr with different Bi contents.

Figure S20. XRD pattern of 2% Au-BiOBr composites.

Figure S21. XRD pattern of 2% Bi-TiO_2.

Figure S22. Cyclic voltammetry (CV) curve of 2% Bi-BiOBr shows the reduction of BiOBr to metallic Bi at cathodic potentials.

Figure S23. Calibration curve used for the qualification of N_2H_4.

Figure S24. The content of N_2H_4 before and after photocatalytic nitrogen fixation experiment over 2% Bi-BiOBr.

Figure S25. Concentrations of produced NO_3^- and NO_2^- over 2% Bi-BiOBr under visible light irradiation (≥ 420 nm).

Table S1 Surface areas of bare BiOBr and Bi/BiOBr composites based on N_2 adsorption-desorption measurements.

Table S2 FWHWs of the Bi (012) diffraction peak in Bi/BiOBr composites.

Table S3 Recent studies on photocatalytic reduction of nitrogen over bismuth-based photocatalysts.

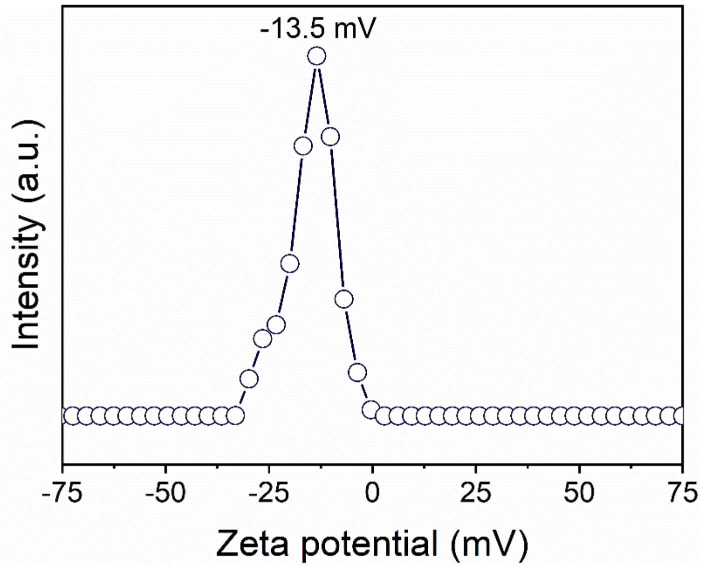

Figure S1. Zeta potential of bare BiOBr.

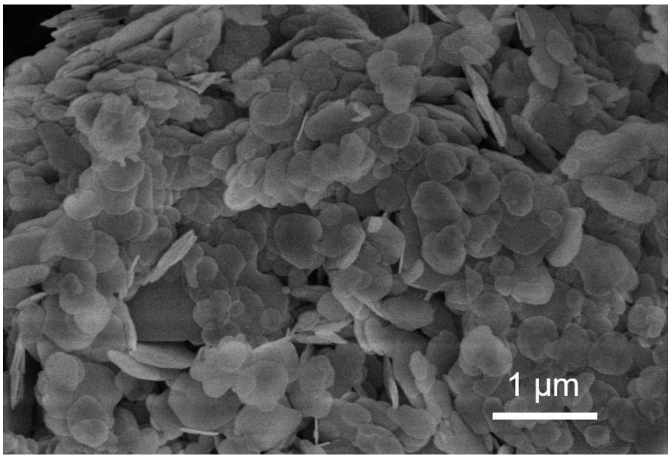

Figure S2. SEM image of bare BiOBr.

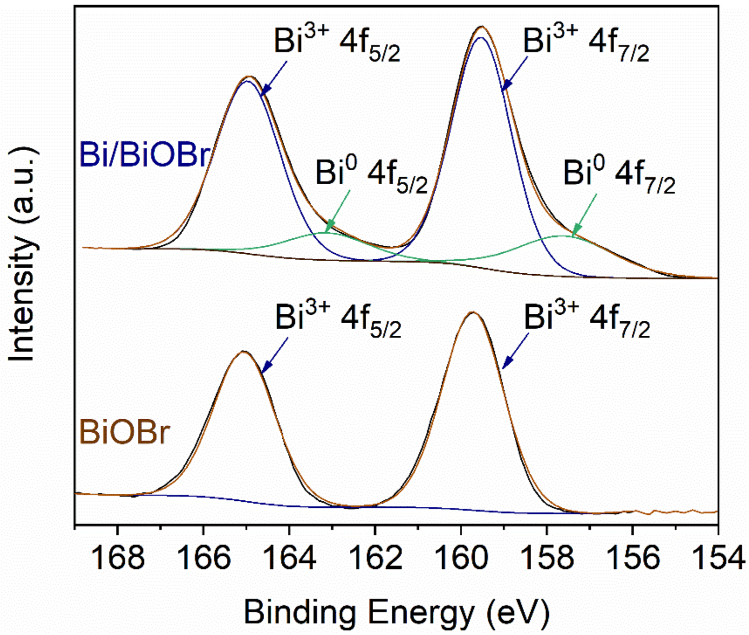

Figure S3. Fitted XPS Bi 4f spectra of the as-prepared bare BiOBr and Bi/BiOBr composites.

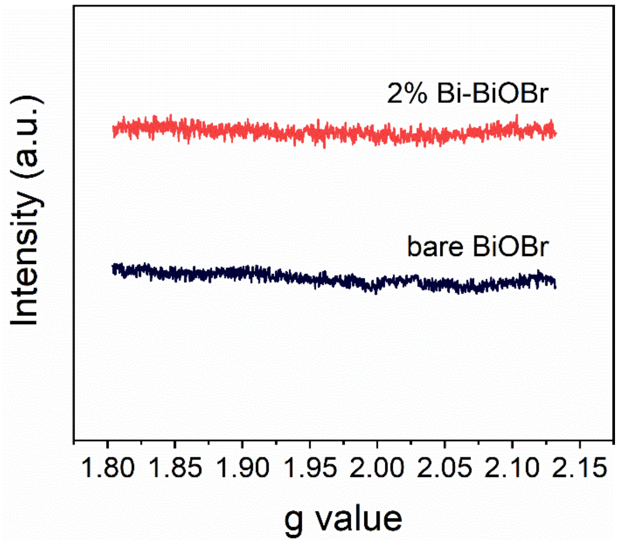

Figure S4. ESR spectra of bare BiOBr and 2% Bi-BiOBr.

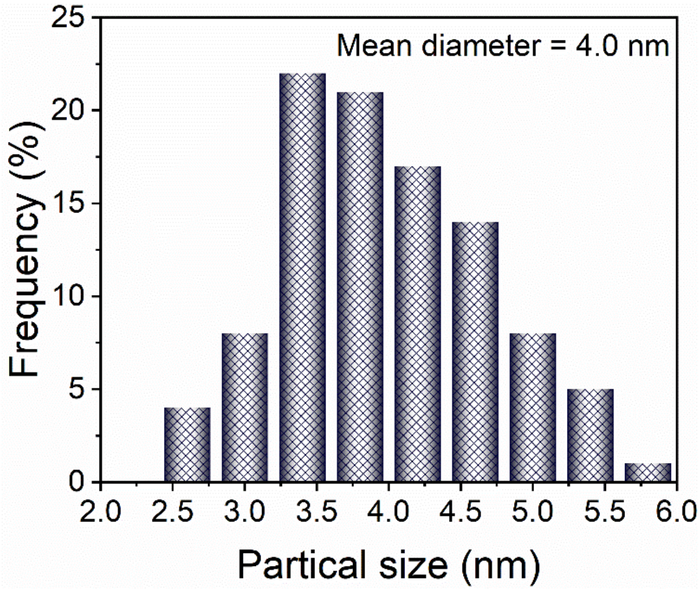

Figure S5. Size distribution histogram of Bi nanoparticles.

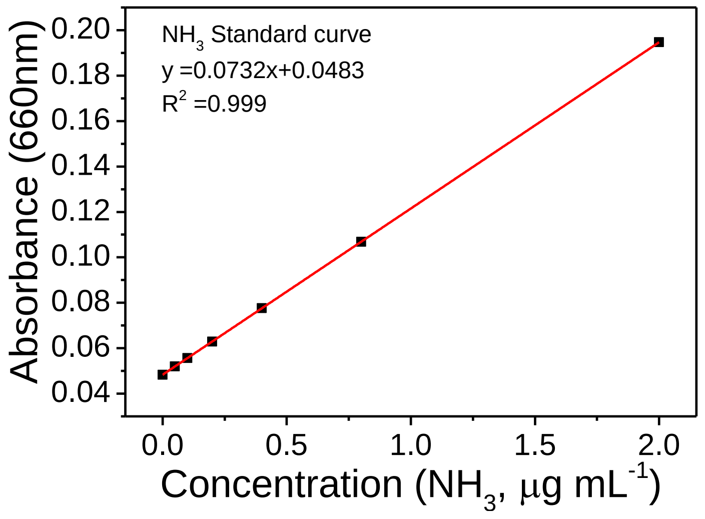

Figure S6. Calibration curve used for the qualification of NH_4^+ by indophenol blue method.

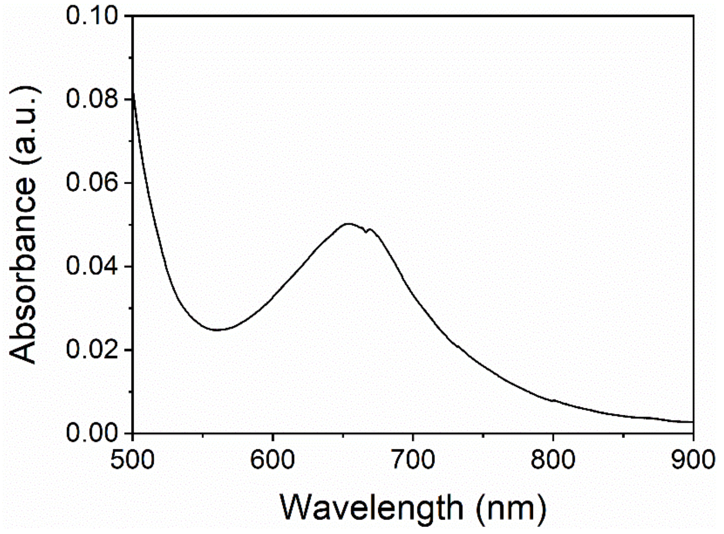

Figure S7. Spectrophotometric assay of the background ammonia by the indophenol blue method.

***Supplementary Note 1*:** Previous studies have demonstrated that commercial $^{15}N_2$ supplies can be contaminated with $^{15}NO_x$ as high as 0.1 mol%[1], while reduction of NO_x is both thermodynamically and kinetically more facile than that of N_2[2]. This is more than sufficient to provide the reported rates of nitrogen reduction reaction[3]. In this circumstance, the $^{15}N_2$ isotope study for the investigation on the catalytic

reduction of N_2 is not completely reliable unless addressing the $^{15}NO_x$ contamination problem. Noteworthily, N_2 with ultrahigh purity (99.999%) has been used in our experiments. The detected impurities include O_2 (1.8 ppm), H_2 (0.1 ppm), CO (0.4 ppm), CO_2 (0.1 ppm), and H_2O (0.7 ppm), which have no interference with the analysis of the nitrogen source in the produced NH_3. In addition, previous study demonstrates that Au catalyst is superior to Bi for the catalytic reduction of NO_x to ammonia[3]. Notably, in our investigation, 5.6 μg of ammonia has been detected over the Bi/BiOBr photocatalyst, which is 7 times as high as that for Au/BiOBr. This result suggests that NO_x should not be the source for the production of ammonia in our experimental system. On account of this analysis and in combination with the results of the control experiments, it is reasonable to conclude that the detected ammonia mainly originates from N_2 reduction in the present investigation scheme.

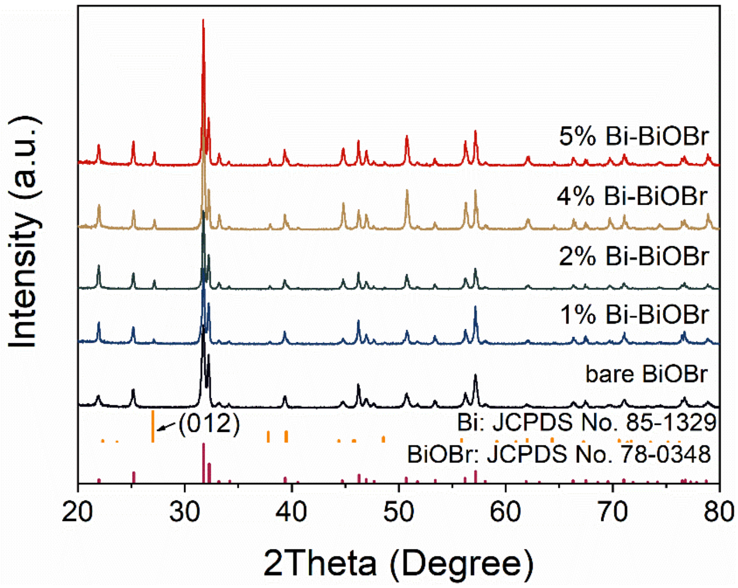

Figure S8. XRD patterns of bare BiOBr and Bi/BiOBr composites with different Bi ratios.

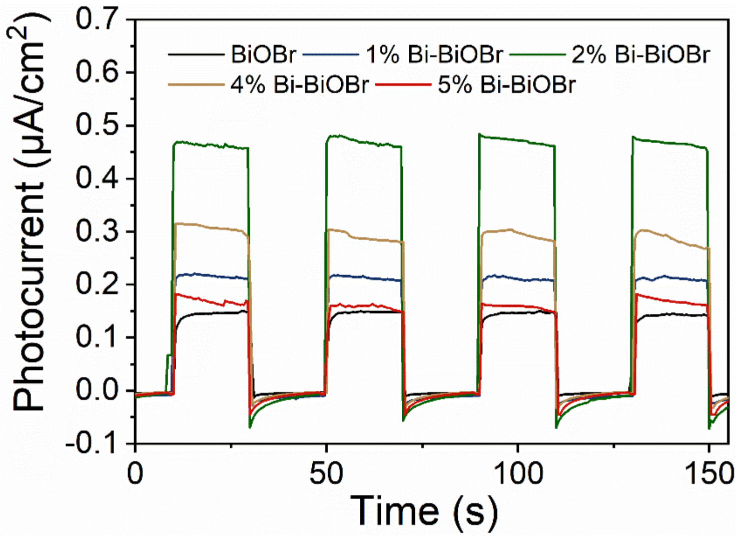

Figure S9. Transient photocurrent responses of the Bi/BiOBr composites under visible light irradiation (λ ≥ 420 nm) without bias.

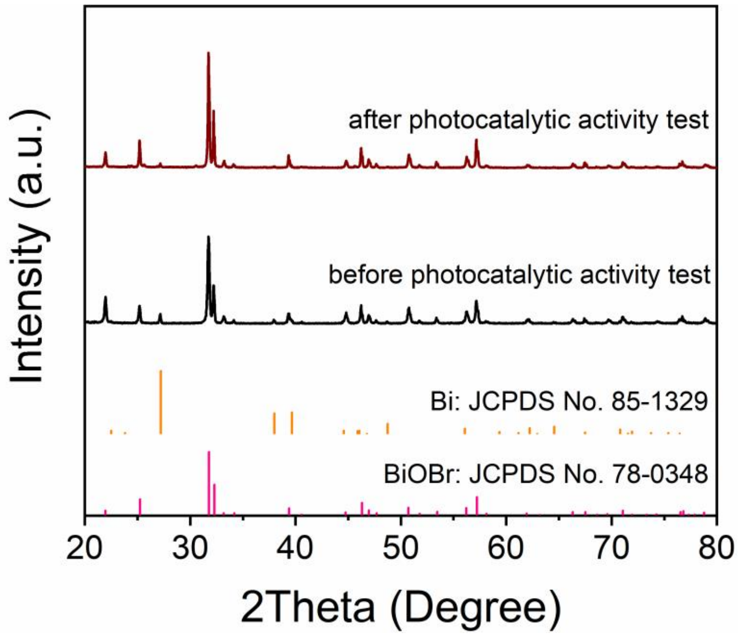

Figure S10. XRD patterns of 2% Bi-BiOBr composite before and after photocatalytic activity test.

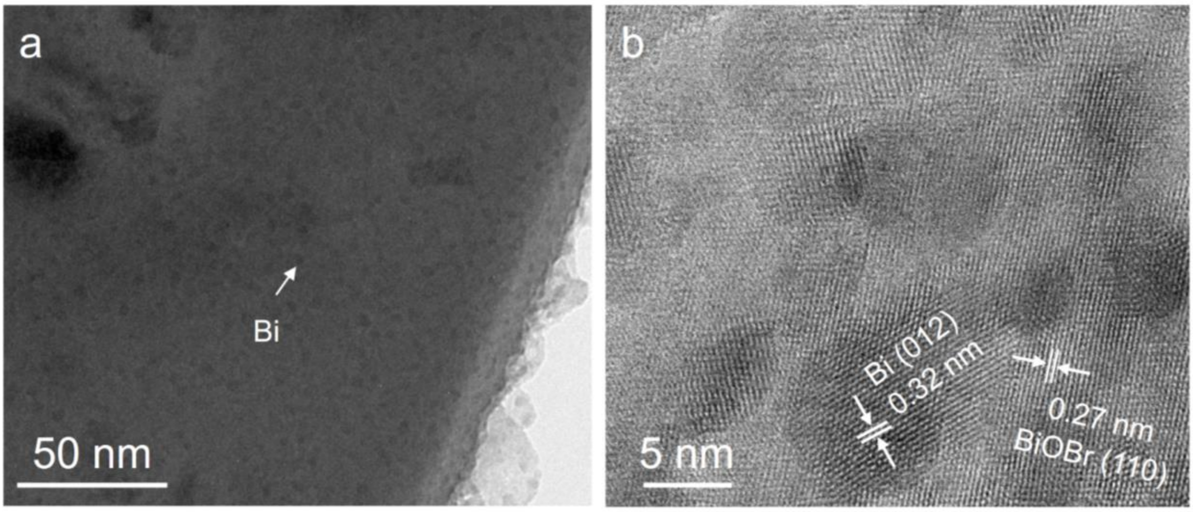

Figure S11. (a) TEM and (b) HRTEM images of 2% Bi-BiOBr after 5 cycles of photocatalytic reaction.

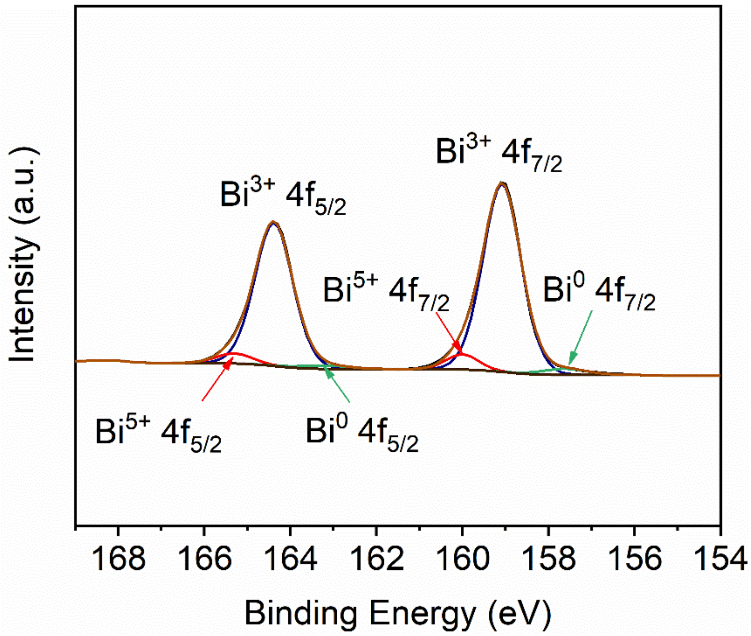

Figure S12. Fitted XPS Bi 4f spectra of 2% Bi-BiOBr composites after photoactivity test.

***Supplementary Note 2*:** According to the XPS result, it is found that the Br/Bi ratio of the used Bi/BiOBr photocatalysts decreased to 0.42 compared to that of the fresh

sample before the photocatalytic N_2 reduction (0.59), indicating the loss of lattice Br during photocatalysis. Similar loss of the halide species has also been observed for another layered bismuth oxyhalide of BiOCl due to the low electronegativity of Cl[4]. Analogously, the consumption of Br can be ascribed to the oxidation of Br^- by photogenerated holes (h^+) according to equation (1):

$$2Bi^{III}OBr + 2h^+ \rightarrow 2Bi^{III}O^+ + Br_2 \quad (1)$$

The photooxidation of lattice Br by h^+ in the presence of hole scavengers in the present system suggests that the transfer of photogenerated h^+ from the bulk of photocatalysts to the surface is sluggish, which has also been revealed previously[5].

The consumption of lattice Br leads to a loss of charge balancing, resulting in a 0.6 eV red shift of the Bi^{3+} binding energies to 159.1 ($4f_{7/2}$) and 164.4 eV ($4f_{5/2}$) (**Figure S7**). Furthermore, Bi^{5+} species have been also detected due to the subsequent self-oxidation according to equation (2):

$$2Bi^{III}O^+ + 2H_2O + 2h^+ \rightarrow Bi^{II}Bi^{V}O_4 + 4H^+ \quad (2)$$

Based on the XPS results, it is speculated that the photoactivity decrease of Bi/BiOBr for N_2 reduction is ascribed to the photocorrosion of the BiOBr substrate. Further loading of cocatalysts shuttling photogenerated h^+ could be useful to mitigate the deactivation of Bi/BiOBr photocatalysts.

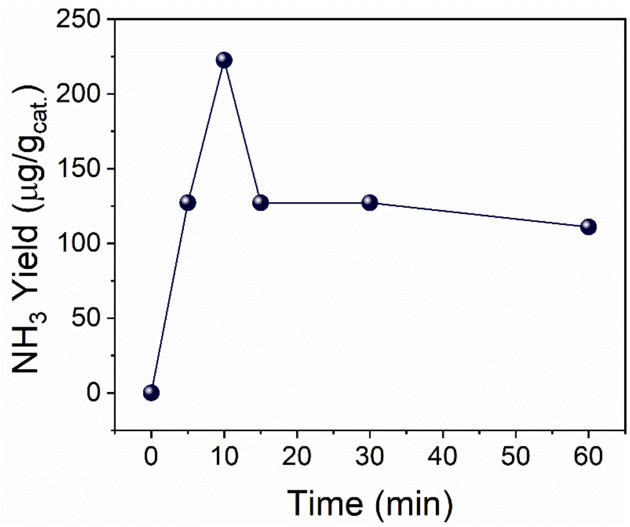

Figure S13. Time dependent NH_3 yield profile of 2% Bi-BiOBr under visible light irradiation ($\lambda \geq 420$ nm) in N_2 atmosphere.

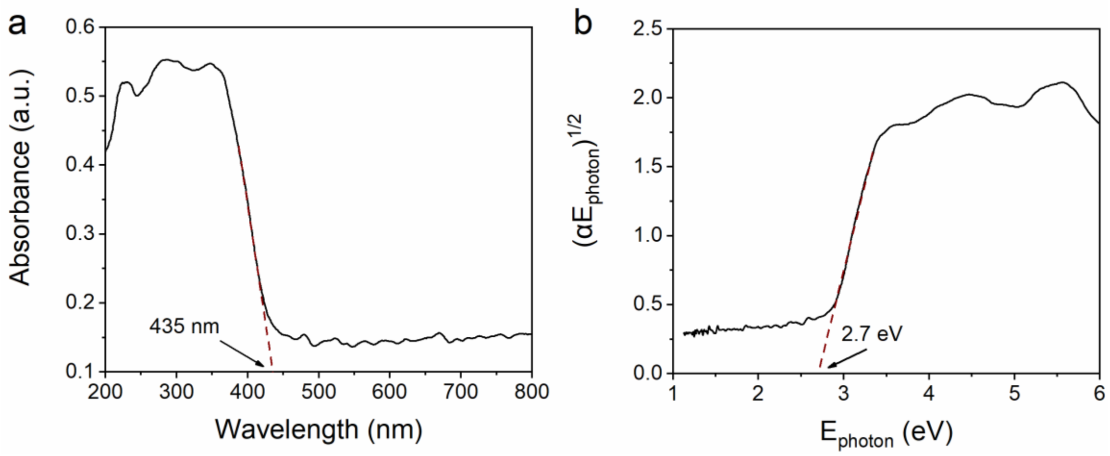

Figure S14. (a) DRS spectrum and (b) Tauc plot of $(\alpha h\nu)^{1/2}$ versus photon energy of bare BiOBr.

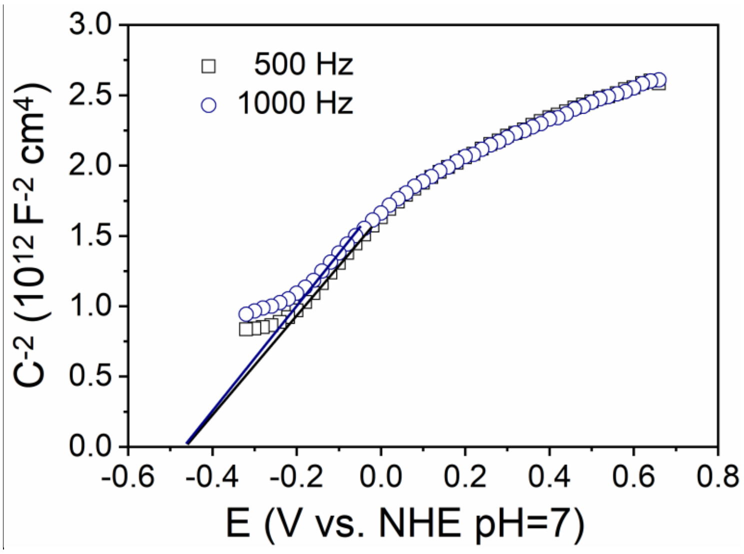

Figure S15. Mott–Schottky (MS) plots of BiOBr.

***Supplementary Note 3*:** It is known that when a semiconductor electrode contacts with an electrolyte solution, electrons will transfer across the semiconductor/electrolyte interface until the Fermi levels of electrons in the solid and the solution are equalized. The interfacial electron transfer generates a space charge

layer in the semiconductor, and conduction and valence band edges are bent such that a potential barrier is established against further electron transfer across the interface[6]. Changing the voltage of the semiconductor artificially through the use of a potentiostat causes the separation of Fermi levels of the semiconductor and the electrolyte. The level of band bending owing to electron depletion in the semiconductor will change depending on the externally applied voltage[7]. When a certain voltage is applied, there is no band bending and the net rate of electron transfer across the interface is zero. Under such situation, the semiconductor is at its flatband potential, which equals the Fermi level of the semiconductor[6]. Therefore, the Fermi level of semiconductor can be determined by experimentally measuring the flatband potential based on Mott-Schottky plots[8].

The relationship of Fermi level (E_F) and conduction band minimum (E_C) of n-type semiconductors can be described by the following equation[9]:

$$E_C - E_F = kTln\left(\frac{N_C}{n}\right)$$

where k is Boltzmann constant, N_C is effective density of states in conduction band, and n is concentration of free electron. At medium temperatures (ca. 100 to 500 K), n is equivalent to N_C[10], and thus conduction band minimum of n-type semiconductor is approximately equal to its Fermi level.

On the basis of the above analysis, the measured flatband potential according to Mott–Schottky (MS) plots can be used as conduction band minimum of n-type semiconductors, which has been widely adopted in the literatures[11].

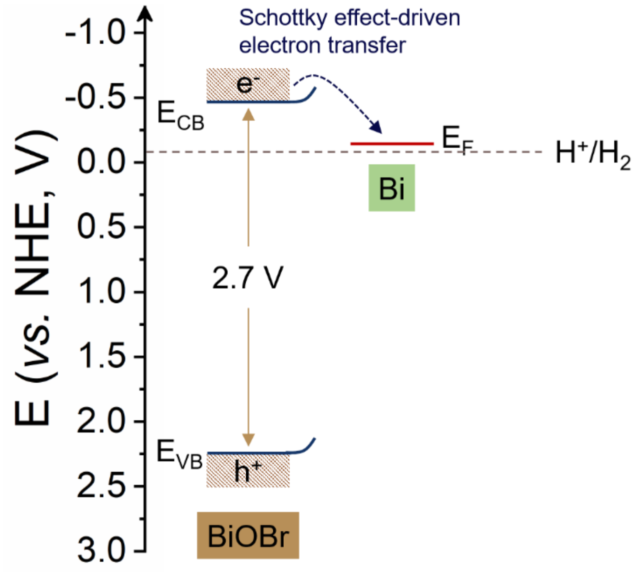

Figure S16. Schematic illustration of electron transfer for Bi/BiOBr composites; CB and VB are short for conduction band and valence band, respectively.

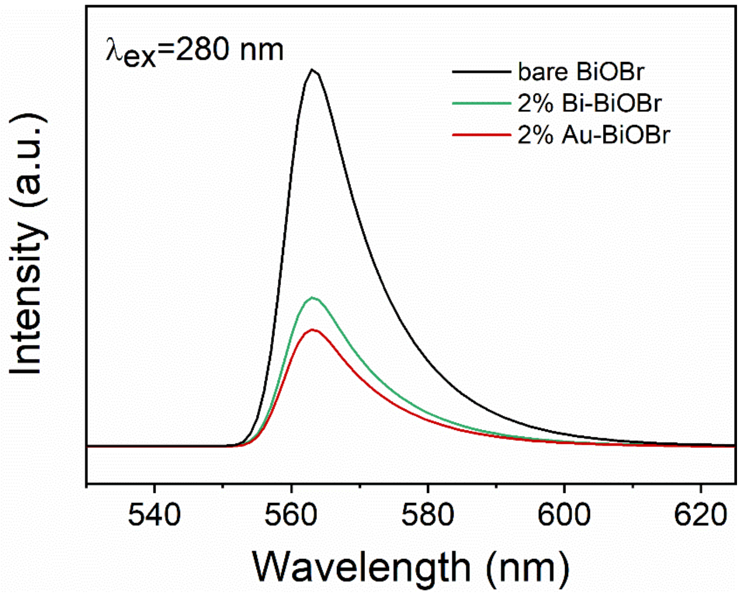

Figure S17. PL spectra of bare BiOBr, 2% Bi-BiOBr, and 2% Au-BiOBr.

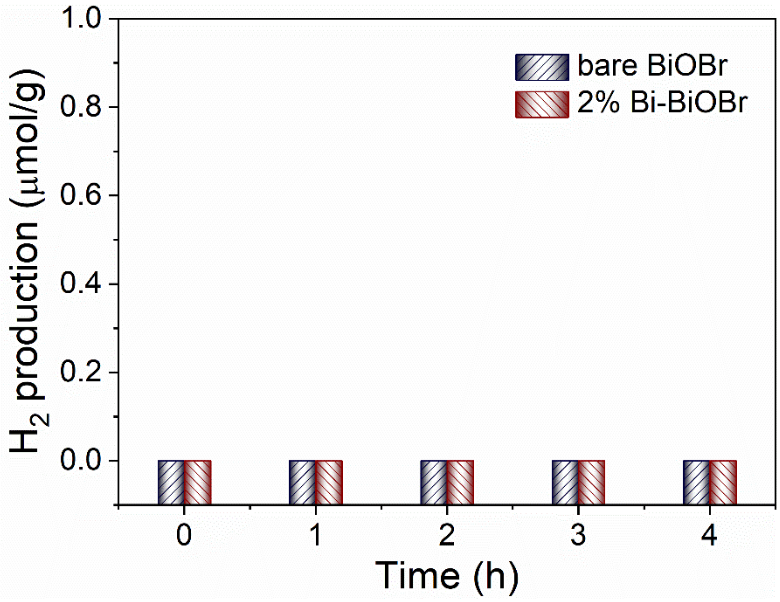

Figure S18. Photocatalytic hydrogen evolution over bare BiOBr and 2% Bi-BiOBr under visible light irradiation (≥ 420 nm).

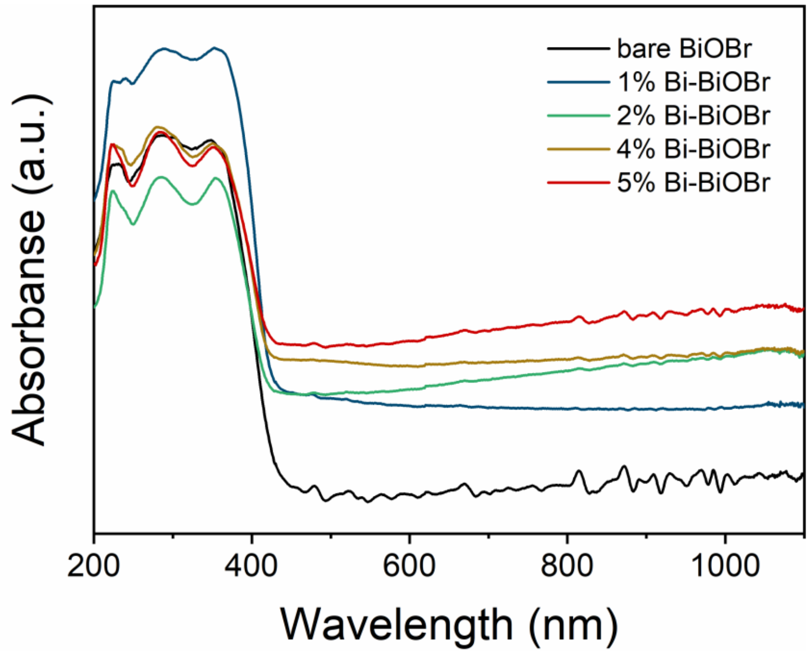

Figure S19. DRS spectra of bare BiOBr and Bi/BiOBr with different Bi contents.

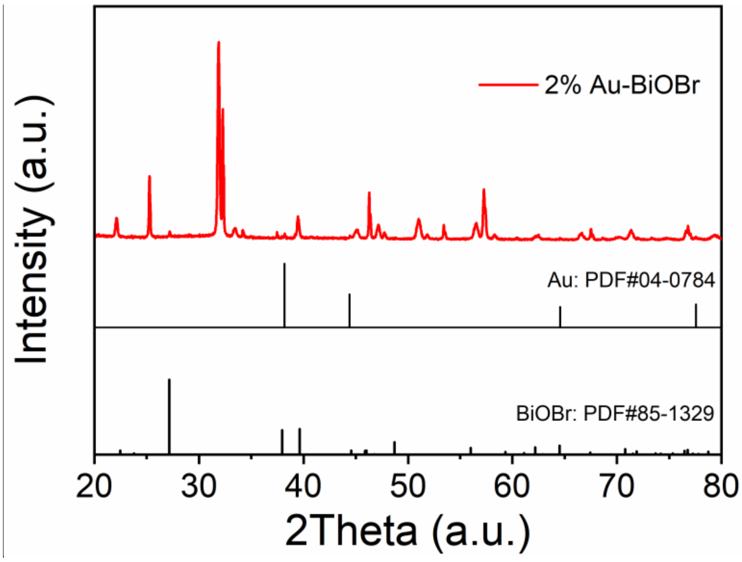

Figure S20. XRD pattern of 2% Au-BiOBr composites.

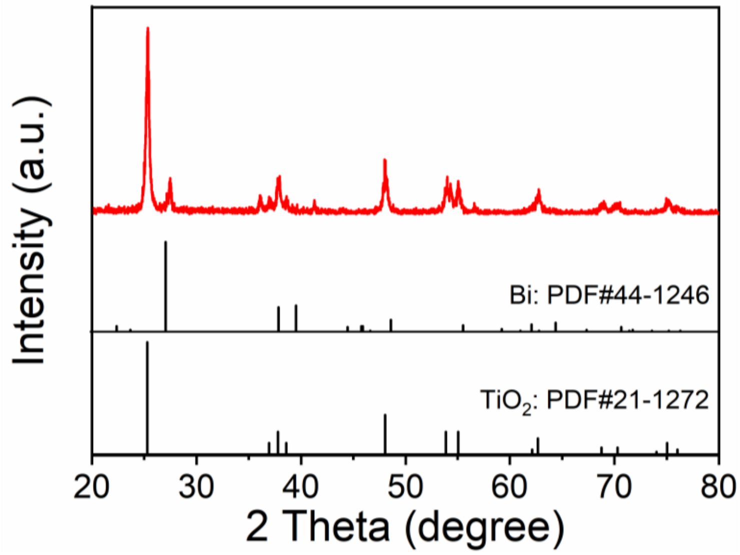

Figure S21. XRD pattern of 2% Bi-TiO_2.

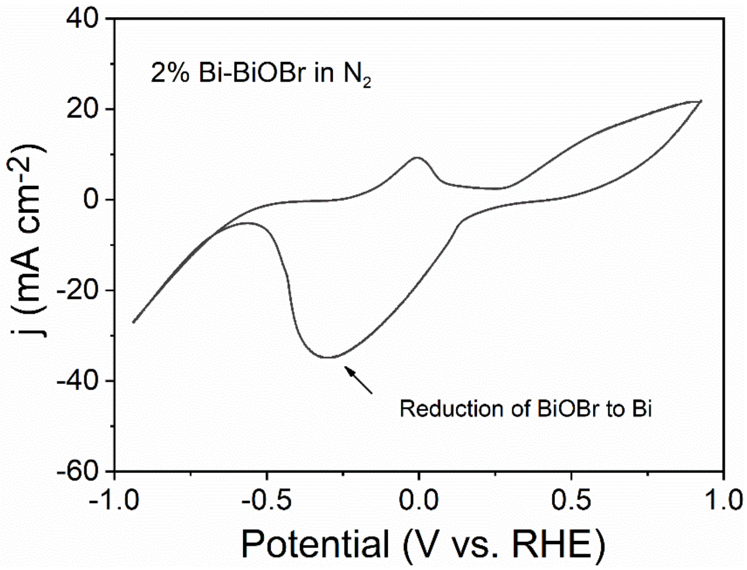

Figure S22. Cyclic voltammetry (CV) curve of 2% Bi-BiOBr shows the reduction of BiOBr to metallic Bi at cathodic potentials.

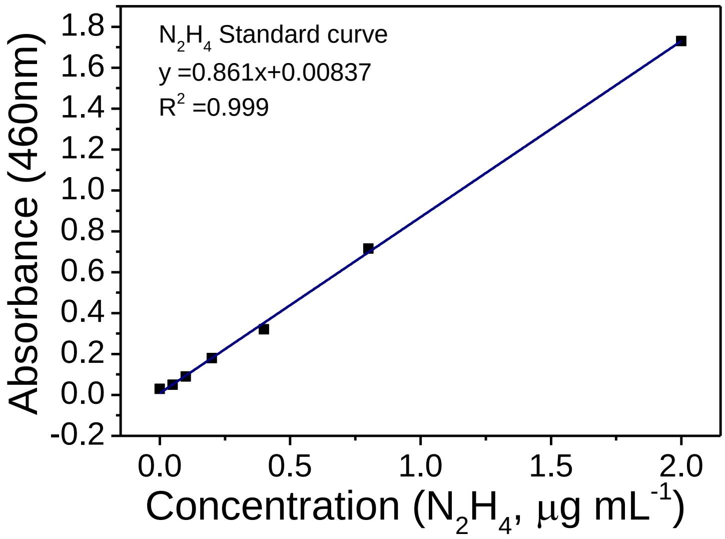

Figure S23. Calibration curve used for the qualification of N_2H_4.

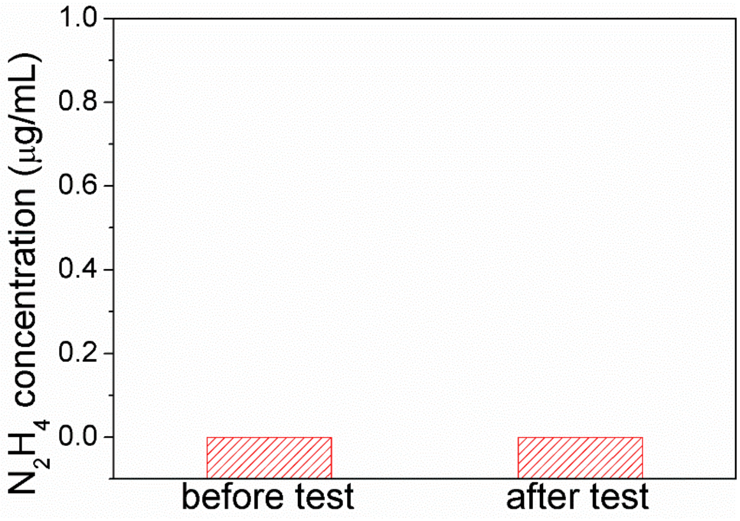

Figure S24. The content of N_2H_4 before and after photocatalytic nitrogen fixation experiment over 2% Bi-BiOBr.

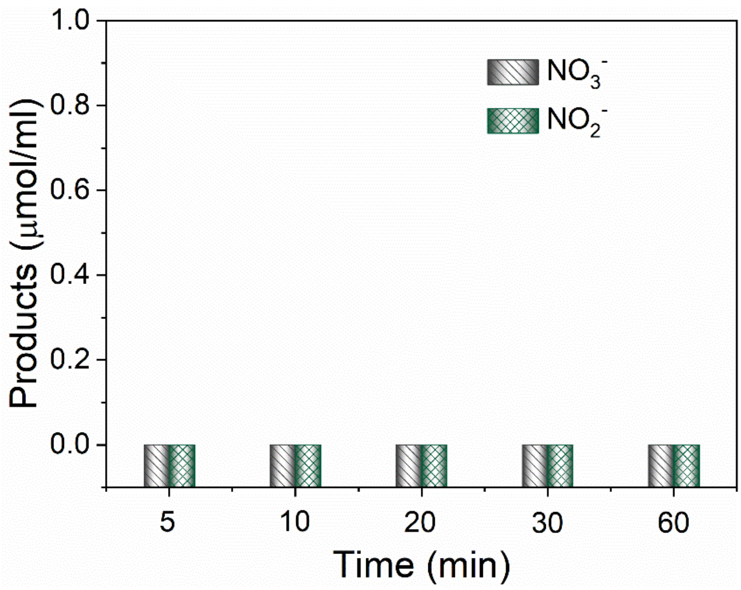

Figure S25. Concentrations of produced NO_3^- and NO_2^- over 2% Bi-BiOBr under visible light irradiation (≥ 420 nm).

Table S1 Surface areas of bare BiOBr and Bi/BiOBr composites based on N_2 adsorption-desorption measurements.

Sample	S_{BET} (m^2 g^{-1})
BiOBr	10.6
1% Bi-BiOBr	4.2
2% Bi-BiOBr	5.6
4% Bi-BiOBr	3.2
5% Bi-BiOBr	5.9

Table S2 FWHWs of the Bi (012) diffraction peak in Bi/BiOBr composites.

Sample	FWHW
1% Bi-BiOBr	0.14°
2% Bi-BiOBr	0.14°
4% Bi-BiOBr	0.13°
5% Bi-BiOBr	0.13°

Table S3 Recent studies on photocatalytic reduction of nitrogen over bismuth-based photocatalysts.

Catalysts	Light source, scavenger	Ammonia production rate [mg h^{-1} g_{cat}^{-1}]	Reference
Bi-BiOBr	≥ 420 nm, Na_2SO_3	1.336	This work
BiOCl-001/BiOCl-010	Full spec., methanol	0.405/1.57	[12]
BiOBr-001-OVs	Full spec., /	3.796	[13]
Bi_3O_4Br	Full spec., /	0.85	[14]
Fe-doped Bi_2MoO_6	> 400 nm, /	1.7	[15]
Br-doped BiOCl-OV	> 400 nm, /	2.142	[16]
Au-$(BiO)_2CO_3$	Full spec., /	0.6494	[17]
Ag-δ-Bi_2O_3	> 400 nm, /	0.17	[18]
$ZnIn_2S_4$-BiOCl	≥ 420 nm, /	0.2482	[19]
GQD-Bi_2WO_6	Full spec., ethanol	0.819	[20]
Bi_2WO_6-PAN	≥ 420 nm, /	2.38	[21]
Bi_2MoO_6-OV-BiOBr	Full spec., /	1.54	[22]
Ag-AgI-δ-Bi_2O_3	> 420 nm, /	1.428	[23]
Ag-AgBr-$Bi_4O_5Br_2$	Full spec., methanol	0.305	[24]
β-Bi_2O_3-BiOCl	Full spec., methanol	3.49	[25]

References

[1] a) R. Dabundo, M. F. Lehmann, L. Treibergs, C. R. Tobias, M. A. Altabet, P. H. Moisander, J. Granger, *PLoS One* **2014**, *9*, e110335; b) V. Moreira-Coello, B. Mouriño-Carballido, E. Marañón, A. Fernández-Carrera, M. PÉrez-Lorenzo, A. Bode, *J. Plankton Res.* **2019**, *41*, 567-570.

[2] a) A. C. A. De Vooys, M. T. M. Koper, R. A. Van Santen, J. A. R. Van Veen, *J. Catal.* **2001**, *202*, 387-394; b) J. Martínez, A. Ortiz, I. Ortiz, *Appl. Catal. B: Environ.* **2017**, *207*, 42-59.

[3] J. Choi, H.-L. Du, C. K. Nguyen, B. H. R. Suryanto, A. N. Simonov, D. R. MacFarlane, *ACS Energy Lett.* **2020**, *5*, 2095-2097.

[4] Y. Shiraishi, M. Hashimoto, K. Chishiro, K. Moriyama, S. Tanaka, T. Hirai, *J. Am. Chem. Soc.* **2020**, *142*, 7574-7583.

[5] a) T. Simon, N. Bouchonville, M. J. Berr, A. Vaneski, A. Adrović, D. Volbers, R. Wyrwich, M. Döblinger, A. S. Susha, A. L. Rogach, F. Jäckel, J. K. Stolarczyk, J. Feldmann, *Nat. Mater.* **2014**, *13*, 1013-1018; b) K. Wu, Z. Chen, H. Lv, H. Zhu, C. L. Hill, T. Lian, *J. Am. Chem. Soc.* **2014**, *136*, 7708-7716.

[6] Y. Xu, M. A. A. Schoonen, *Am. Mineral.* **2000**, *85*, 543-556.

[7] K. Gelderman, L. Lee, S. W. Donne, *J. Chem. Educ.* **2007**, *84*, 685.

[8] A. J. Nozik, *Annu. Rev. Phys. Chem.* **1978**, *29*, 189-222.

[9] S. M. Sze, K. K. Ng, *Physics of semiconductor devices*, John wiley & sons, **2006**.

[10] S. Robert Allan, *Semiconductors. 2nd ed.*, Cambridge University Press: London, **1979**.

[11] a) J. Zhang, X. Chen , K. Takanabe, K. Maeda, K. Domen, J. D. Epping, X. Fu, M. Antonietti, X. Wang *Angew. Chem. Int. Ed.* **2010**, *49*, 441-444; b) J. Ran, G. Gao, F.-T. Li, T.-Y. Ma, A. Du, S.-Z. Qiao, *Nat. Comm.* **2017**, *8*, 13907; c) L. Yang, G. Dong, D. L. Jacobs, Y. Wang, L. Zang, C. Wang, *J. Catal.* **2017**, *352*, 274-281; d) Y. Zhou, L. Zhang, W. Wang, *Nat. Comm.* **2019**, *10*, 506.

[12] H. Li, J. Shang, J. Shi, K. Zhao, L. Zhang, *Nanoscale* **2016**, *8*, 1986-1993.

[13] H. Li, J. Shang, Z. Ai, L. Zhang, *J. Am. Chem. Soc.* **2015**, *137*, 6393-6399.

[14] J. Di, J. Xia, M. F. Chisholm, J. Zhong, C. Chen, X. Cao, F. Dong, Z. Chi, H. Chen, Y. X. Weng, J. Xiong, S. Z. Yang, H. Li, Z. Liu, S. Dai, *Adv. Mater.* **2019**, e1807576.

[15] Q. Meng, C. Lv, J. Sun, W. Hong, W. Xing, L. Qiang, G. Chen, X. Jin, *Appl. Catal. B: Environ.*, **2019**, *256*, 117781.

[16] D. Wu, R. Wang, C. Yang, Y. An, H. Lu, H. Wang, K. Cao, Z. Gao, W. Zhang, F. Xu, *J. Colloid Interface Sci.* **2019**, *556*, 111-119.

[17] C. Xiao, H. Hu, X. Zhang, D. R. MacFarlane, *ACS Sust. Chem. Eng.,* **2017**, *5*, 10858-10863.

[18] X. Gao, Y. Shang, L. Liu, F. Fu, *J. Colloid Interface Sci.* **2019**, *533*, 649-657.

[19] L. Guo, X. Han, K. Zhang, Y. Zhang, Q. Zhao, D. Wang, F. Fu, *Catalysts* **2019**, *9*, 729.

[20] T. Fei, L. Yu, Z. Liu, Y. Song, F. Xu, Z. Mo, C. Liu, J. Deng, H. Ji, M. Cheng, *J. Colloid Interface Sci.* **2019**, *557*, 498-505.

[21] C. Zhang, G. Chen, C. Lv, Y. Yao, Y. Xu, X. Jin, Q. Meng, *ACS Sust. Chem. Eng.*, **2018**, *6*, 11190-11195.

[22] X. Xue, R. Chen, C. Yan, Y. Hu, W. Zhang, S. Yang, L. Ma, G. Zhu, Z. Jin, *Nanoscale* **2019**, *11*, 10439-10445.

[23] X. Gao, Y. Shang, K. Gao, F. Fu, *Nanomaterials* **2019**, *9*, 781.

[24] Y. Chen, C. Zhao, S. Ma, P. Xing, X. Hu, Y. Wu, Y. He, *Inorg. Chem. Front.*, **2019**, *6*, 3083-3092.

[25] J. Shang, H. Chen, B. Zhao, F. Zhou, H. Zhang, X. Wang, *J. Mater. Sci.: Mate. Electron.*, **2019**, *30*, 17956-17962.
